# Legal issues in digital oral health: a scoping review

**DOI:** 10.1186/s12913-023-10476-w

**Published:** 2024-01-03

**Authors:** Rodrigo J. Mariño, Carlos Zaror

**Affiliations:** 1 Center for Research in Epidemiology, Economics and Oral Public Health (CIEESPO), Faculty of Dentistry, Universidad de La Frontera, Temuco, Chile; 2 Melbourne Dental School, University of Melbourne, Melbourne, Australia; 3 Department of Pediatric Dentistry and Orthodontics, Faculty of Dentistry, Universidad de La Frontera, Temuco, Chile

**Keywords:** Oral health, Legal issues, Teledentistry, Digital Health

## Abstract

**Background:**

This scoping review aims to systematically and critically describe the numerous legal challenges brought about by the utilization of digital oral health in the delivery of oral healthcare.

**Methods:**

A systematic search was conducted. The following electronic databases were reviewed from inception up to March 2023: MEDLINE, Embase, Scopus, and LILACS. The search included any scientific document and paper in English, Spanish, or Portuguese on legal issues raised using digital health in oral healthcare delivery. Two reviewers conducted the selection process and data extraction. Legal issues raised concerning the adoption of digital health technology were analysed using the modified Mars’ framework.

**Results:**

Seventeen studies were included. Most of the documents identified and covered generic aspects of delivering digital oral healthcare (n = 11) without explicitly referring to any dental specialty. The most mentioned legal issues were data security (n = 15); liability and malpractice (n = 14); consent (n = 12); and confidentiality (n = 12). To a lower extent, patient-practitioner relationship (n = 11); and license and jurisdiction (n = 11) were also covered. These were followed by privacy of information (n = 10); adequacy of records (n = 9); and e-referrals (n = 8). On the other hand, fewer studies commented on social media use (n = 3), authentication (n = 2); or e-prescriptions (n = 2). Before implementing any digital health solution, practitioners need to be aware of the many legal issues that the introduction of these technologies involves, be clear where the responsibility lies, and apply extreme caution in following national guidelines. Current literature concentrates on a few well-known legal issues. Issues around authentication, use of social media, and e-prescriptions received less attention.

## Introduction

Information and Communication Technology (ICT) and digital health technology are changing the way health professionals work, acquire knowledge and training, and communicate with patients as well as their professional networks. Digital health has strengthened the delivery of dental healthcare through improving access, monitoring, sharing, and use of quality data. In recent years, digital health has been successfully implemented in oral healthcare practice and appears to be a promising tool in the management of patients requiring non-surgical and surgical treatment, especially by reducing costs and waiting times [1–3]. Digital health is not a single, uniform type of technology. Digital health extends the concept of e-health to include a wider range of devices, Apps, software and equipment [4]. It also encompasses technologies such as artificial intelligence and robotics and data analytics to improve the health and well-being of patients, and/or to reduce the costs of services [5].

Along with its benefits, the rapidly evolving environment of digital health technology raises numerous challenges regarding ethical, social, legal, policy, and funding concerns, which may act as barriers to its adoption [6, 7]. These challenges include quality of care, compensation, liability, safety, usability, privacy concerns, equity, and accessibility [6]. There has been a general lack of discussion of the broad legal issues involved [7].

Since its early implementation, jurisdictions have developed guidelines about the use of digital health. Digital health as a discipline is covered by several specific laws relating to health, telecommunications, information and communication, data sensitivity and privacy. More specifically, tele(oral)health providers are subject to regulation by their national professional boards and other regulatory bodies (e.g., Australian Health Practitioner Regulation Agency). Countries including Australia [8–10]; Brazil [11]. Canada [7, 12]; Germany [13]; and the USA [14] have regulations about the legal and ethical implications of digital oral health as well as standards for e-Health Records (EHR) [15]. Quality assessment is also provided by the governmental organisations that oversee and assess the quality of products available in the health market (e.g., Therapeutic Goods Administration in Australia [16]; Federal Drug Administrating (FDA) in USA, etc.). Professional organisations also ensure quality, furthermore, they have a critical role to play in the advancement of digital health technology by identifying knowledge gaps and defining standards.

This scoping review aims to describe in a systematic and critical manner, the numerous legal and policy issues and challenges that the utilization of digital oral health brings about in the context of oral healthcare. By identifying current and expected legal challenges, we aim to increase awareness and contribute to a better understanding of the factors that may determine successful (or failed) implementation of digital health in the oral healthcare field, dental education and in future oral healthcare practice. In doing so, this manuscript is not intended to provide legal advice or to be a comprehensive text on regulatory issues around digital oral health; rather, the emphasis will be on identifying general best practices and providing examples to raise awareness and explore the legal, and regulatory issues posed by tele oral healthcare.

## Methodology

### Design and eligibility criteria

To identify what legal issue have been examined and discussed in the context of digital oral healthcare delivery, a scoping review was conducted [17]. A scoping review approach was chosen because it allowed for synthesizing information from diverse sources, including theoretical reviews, and qualitative as well as quantitative research. Therefore, we included any scientific document or paper in English, Spanish or Portuguese, reporting information that answers our research question. Studies assessing only technical and engineering aspects of teledentistry, letters to editors and abstracts of conferences were excluded.

### Sources of information and search strategy

A systematic search of the literature was conducted using the following electronic databases: MEDLINE, Embase, LILACS and Scopus, from inception to December 2022. Reference listings of selected articles were hand searched to identify other possible studies. The details of the search strategy used are given in Table 1. Additional articles were also identified through the backward snowballing technique, but gray literature [information not published through traditional academic channels, such as peer-reviewed journals or books], was not systematically reviewed.

**Table 1:** Search strategy used on each database

Source	Strategy
Medline	(((((((((((((((((((((((((((malpractice) OR (“Malpractice“[Mesh])) OR (ethics)) OR (bioethics)) OR (“Ethics“[Mesh])) OR (“Bioethics“[Mesh])) OR (license)) OR (“quality of care”)) OR (jurisdiction)) OR (Informed consent)) OR (“Informed Consent“[Mesh])) OR (confidentiality)) OR (“Confidentiality“[Mesh])) OR (Privacy)) OR (patient privacy)) OR (data security)) OR (“Computer Security“[Mesh])) OR (Authentication)) OR (“Remuneration“[Mesh])) OR (remuneration)) OR (e-referrals)) OR (“Referral and Consultation“[Mesh])) OR (referral)) OR (consultation)) OR (social media)) OR (“Social Media“[Mesh])) OR (“Quality of Health Care“[Mesh])) AND (teledentistry)
EMBASE	(malpractice OR ‘malpractice’/exp OR ethics OR ‘ethics’/exp OR bioethics OR ‘bioethics’/exp OR license OR jurisdiction OR (quality AND of AND care) OR ‘quality of care’/exp OR (informed AND consent) OR ‘informed consent’/exp OR confidentiality OR ‘confidentiality’/exp OR privacy OR (data AND security) OR ‘information security’/exp OR authentication OR remuneration OR ‘remuneration’/exp OR ‘e referrals’ OR referrals OR ‘consultation’/exp OR consultation OR (social AND media) OR ‘social media’/exp) AND (teledentistry OR ‘teledentistry’/exp) AND [embase]/lim
SCOPUS	(TITLE-ABS-KEY (malpractice OR ethics OR bioethics OR license OR “quality of care” OR jurisdiction OR “informed consent” OR confidentiality OR privacy OR data AND security OR authentication OR remuneration OR e-referrals OR referral OR consultation OR “social media”)) AND (TITLE-ABS-KEY (teledentistry))
LILACS	(dentistry OR oral) AND (teledentistry OR “digital health”)

### Selection of sources of evidence

All references identified were exported into the Research Information Systems file and uploaded into the EndNote software, where duplicates were automatically eliminated. Articles were then exported into the Rayyan online software (https://rayyan.qcri.org) for selection. Two reviewers (RM and CZ) independently performed the selection of the studies by title and abstract, and then by full text according to the eligibility criteria. If there was a discrepancy, a consensus was reached. The reviewers were not blinded to the authors or journals. The reasons for exclusions were recorded.

### Data charting process

One reviewer (RM) extracted relevant data from eligible studies, and an additional reviewer checked the information extracted for accuracy (CZ) (non-independent verification of data extraction). The following items were extracted from each article using a predefined Excel form: study identification information, country, study objective, specialty area of teledentistry, issue of teledentistry considered, and principal conclusions of the study.

The results were categorised following a modified version of Mars’ framework of the legal issues raised in relation to the adoption of digital health technology use, most of them based on telemedicine [18]. According to the framework, issues were categorised as follow:

1. License and jurisdiction of the oral healthcare provider.
2. Quality of care and continuity of care.
3. Patient - practitioner relationship.
4. Consent.
5. Authentication.
6. Confidentiality.
7. Privacy.
8. Data security.
9. e-referrals.
10. Use of social media.
11. Financial and remuneration.
12. Adequacy of records.
13. e-prescriptions.
14. Liability and malpractice.

## Results

### Selection of sources of evidence

**Fig. 1:**
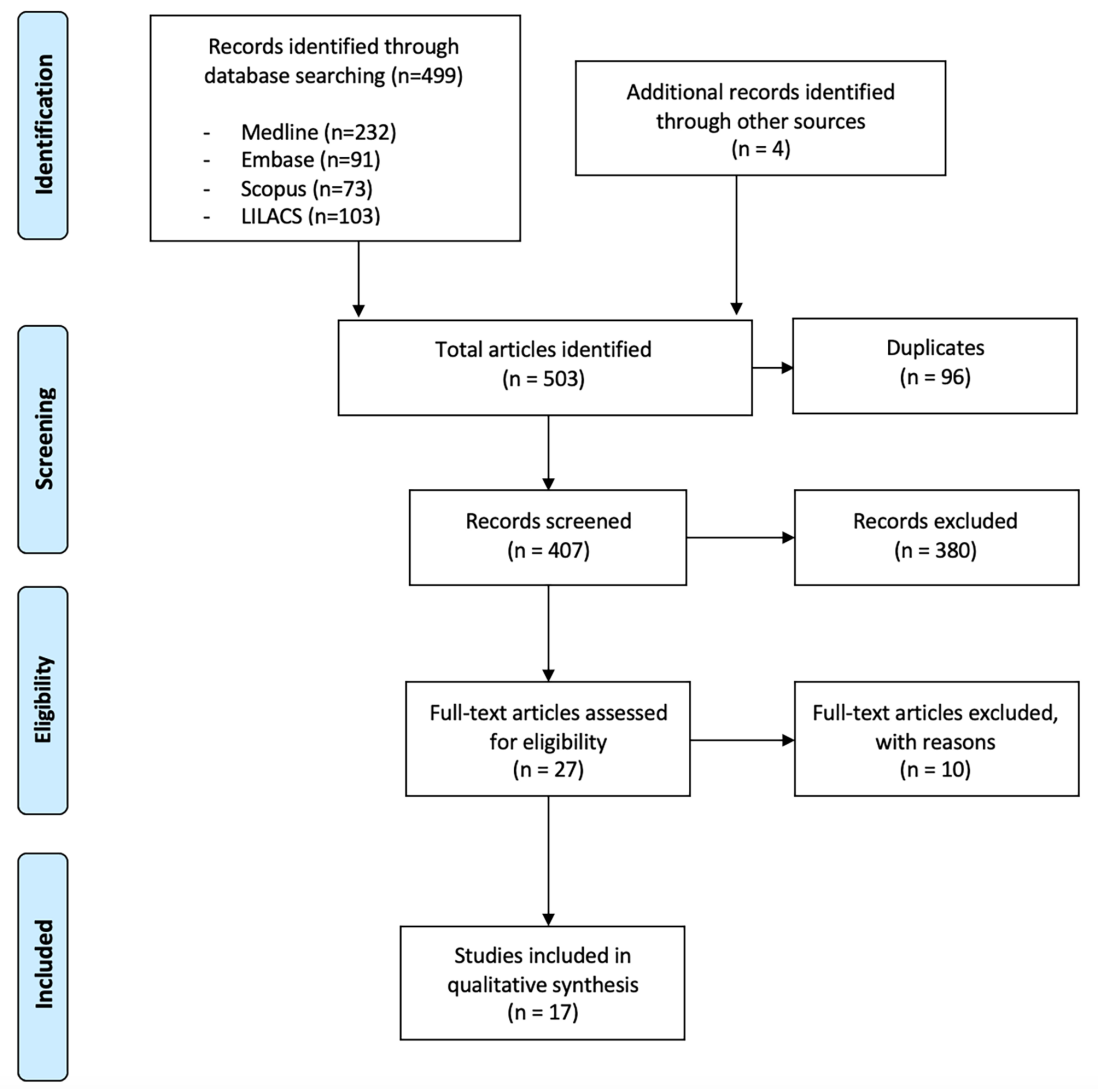
Flow diagram of the scoping review

The search identified a total of 499 citations, and a further four articles were identified by hand search. After duplicates were eliminated, 407 articles were screened by title and abstract, of them, 380 were excluded. A total of 27 full text articles were retrieved and assessed for eligibility. Of these, 10 were excluded because they did not provide an analysis of the issues reviewed in this study. Finally, 17 documents were included. A PRISMA chart (Fig. 1) shows the details of each step of the selection process.

### Characteristics of sources of evidence

Studies included in this review were from seven countries, and two were from multiple countries. The majority were conducted either in the United States of America (n = 5) [19–23; India (n = 4) [19–22]; or Canada (n = 2) [12, 23]. Studies were also conducted in Rumania [1]; Chile [24]; Pakistan [25]; and South Africa [26]. Additionally, two studies included multiple countries [13, 27].

By the type of dental practice (i.e., general practice, dental specialties), the majority were generic and did not explicitly refer to any specialty (n = 11; 64.7%), followed by dental radiology (n = 4; 23.6%) [1,23,27,28,]; orthodontics [1, 28]; and dental prosthetics [1, 22], which were mentioned in two reports each (11.8%). One study mentioned maxillo-facial surgery [1], and another report was specific for pediatric dentistry [27]. Background information and the legal issues considered are summarized in Tables 2 and 3, respectively.

**Table 2:** Characteristics of the included studies

Reference	Country	Specialty area of teledentistry	objective	Main conclusions
Sfikas. 1997 [29]	USA	Not explicit	To review legal and regulatory implications for *teledentistry*	A variety of legal issues in *teledentistry* need to be addressed before it is fully implemented.
Golder et al., 2000 [30]	USA	Not explicit	To examine teledentistry and some of its current legal issues.	Numerous issues require resolution before telemedicine and teledentistry will truly realize their enormous potential to increase access to health care while decreasing health care costs.
McDonald et al., 2007 [23]	Canada	Digital radiology	To discuss the legal impact in oral maxillofacial radiology on dental practice and dental care	Digital health means that dentistry will be held to the accepted standards of the practice of medicine. Careful inquiry into legal issues should be made before the purchase of radiological equipment.
Jampani et al., 2011 [20]	India	Not explicit	To reviews the ethical and legal issues related to the practice of teledentistry and the future of this alternative and innovative method of delivering dental care	A number of issues need to be addressed before teledentistry can reach its potential. Further studies will be required to validate the various aspects of teledental applications
Cartes et al., 2012 [24]	Chile	Not explicit	To review concepts, history, fields of application, legal considerations, and document experiences around the world, as well as to propose some applications and future projections of teledentistry.	Overall, teledentistry was found a positive, overcoming initial clinical and legal questionings.
Peltier et al., 2013 [31]	USA	Not explicit	To review ethical and legal implications of the use of websites, Facebook, review sites, email and other digital innovations in dental practice.	Recent developments in electronic and social media offer exciting possibilities for the enhancement of dental practice, both for patients and practitioners. But there are very real challenges involved, and they pose credible threats to the profession.
Iyer et al., 2013 [21]	India	Not explicit	To review why a dentist should be aware of his/her rights, duties and how the medico-legal claims can be avoided.	Dental practitioners should take proper measures to prevent medicolegal issues in general, but in particular when using digital oral health.
Simon et al., 2016 [32]	USA	Digital radiology	Implementation guidelines and regulatory considerations on the use of *teledentistry* for oral and maxillofacial radiologists	Portability of diagnostic images may make it more difficult to enforce geographic practice boundaries. A national licensure system would be easier to enforce while maintaining high levels of patient safety.
Sykes et al., 2017 [26]	South Africa	Not explicit	To describe the many ethical, legal and professional challenges of using social media in dentistry	Social media can be a powerful and effective tool to improve the quality of health care provision, however, when used thoughtlessly, unethically or illegally it may have negative ramifications for the practitioner as well as the profession.
Bhargava et al., 2019 [19]	India	Not explicit	To review ethics and legal aspects of *teledentistry*	Teledentistry is a relatively new and exciting field with endless potential. clinical guidelines and standards should be established for the practice of *teledentistry.*
Latin-American Association of Paediatric Dentistry 2020 [27]	Latin America	Paediatric dentistry	To provide guidelines for the conduct of virtual paediatric dentistry consultations	
Iorgulescu et al., 2020 [1]	Romania	Prosthodontics, digital radiology, orthodontic, maxillo-facial surgery	Ethics and medico legal challenges are insufficiently addressed. To assess the medico-legal impact of digital health technologies on oral health professionals and patients.	Practitioners should be aware of the ethics and medico legal challenges involved in the use of digital technology in their practices.
Morey et al., 2021 [22]	India	Prosthodontics, digital radiology	To evaluate the medico legal aspects of Teledentistry in everyday practice	Need for further development to be fully implemented
Park et al., 2021 [28]	USA	Orthodontics	To discuss the various types of teledentistry systems for orthodontic practices, implementation guidelines, and important regulation.	Informed consent forms should include teledentistry. Malpractice insurance should also cover for cyber liability insurance increases with teledentistry
Singhal et al., 2021 [12]	Canada	Not explicit	To conduct an environmental scan of all pertinent literature to understand how the government and other organizations have promoted teledentistry and virtual health care since the start of the COVID-19 pandemic.	Although there may be a need for more studies, the observed strengths and upcoming opportunities indicate that teledentistry may fill a major gap in oral health care delivery.
Wolf et al., 2022 [13]	Europe and USA	Not explicit	This review deals with the presentation of the fields of activity of telemedicine in hospitals and in the dental field, with special attention to opportunities and risks.	Access, and delivery of telemedicine applications in dentistry and medicine should be expanded and improved to provide access to all population groups.
Fahim et al., 2022 [25]	Pakistan	Not explicit	Challenges and solutions for *teledentistry* in Pakistan	Government should encourage *teledentistry* and reduce barriers to implement *teledentistry*. Stakeholders should be included in the development of guidelines and regulations.

**Table 3:** Legal issue of teledentistry considered

Reference	License and jurisdiction	Quality and continuity of care	Patient - practitioner relationship	Consent	Authentication	Confidentiality	Privacy	Data security	e-Referrals	Social media	Financial and remuneration	Adequacy of records	e-Prescription	Liability and malpractice
Sfikas, 1997 [29]	✓		✓			✓		✓						✓
Golder et al., 2000 [30]	✓	✓	✓	✓	✓	✓	✓	✓	✓		✓	✓		✓
McDonald et al., 2007* [23]						✓		✓				✓		✓
Jampani et al., 2011* [20]	✓			✓		✓	✓	✓			✓			✓
Cartes et al., 2012 [24]	✓	✓					✓					✓		✓
Peltier et al., 2013 [31]		✓	✓	✓		✓	✓	✓		✓				✓
Iyer et al., 2013 [21]	✓		✓	✓		✓	✓	✓	✓			✓		✓
Simon et al., 2016 [32]	✓													✓
Sykes et al., 2017 [26]			✓	✓		✓	✓	✓		✓				✓
Bhargava et al. 2019 [19]	✓	✓	✓	✓	✓	✓	✓	✓	✓			✓		✓
Latin-American Association of Paediatric Dentistry 2021 [27]			✓	✓		✓		✓	✓			✓	✓	
Iorgulescu et al. 2020 [1]		✓	✓	✓				✓	✓			✓		
Morey et al., 2021 [22]	✓	✓	✓	✓		✓	✓	✓	✓		✓	✓		✓
Park et al., 2021 [28]	✓		✓	✓				✓		✓	✓		✓	✓
Singhal et al., * 2021 [12]	✓			✓		✓	✓	✓	✓		✓	✓		
Wolf et al., 2022* [13]	✓		✓				✓	✓			✓			✓
Fahim et al., 2022* [25]		✓		✓		✓		✓	✓		✓			✓
**Total**	11 (64.1%)	7 (41.2%)	11 (64.7%)	12 (70.6%	2 (11.8%)	12 (70.6%)	10 (58.8%)	15 (88.2%)	8 (47.1%)	3 (17.6%)	7 (41.2%)	9 (52.9%)	2 (11.8%)	14 (82.4%)

The most mentioned legal issues were data security (n = 15; 88.2%); liability and malpractice (n = 14; 82.4%); consent and confidentiality (n = 12; 70.6% each); patient-practitioner relationship, and license and jurisdiction (n = 11; 64.7% each). These issues were followed by privacy of information and adequacy of records (n = 10; 58.8%; and 9; 52.9%, respectively); and e-referrals (n = 8; 47.1%). Particularly low were social media use (n = 3; 17.6%); authentication and e-prescriptions (n = 2; 11.8%, each).

### Synthesis of results

1. Licensure and Jurisdiction. Teledentistry and technology-based consultations are increasingly being used to improve access to health services. The future oral health workforce will enter a world that has largely adapted to the provision of digital oral health care. In the provision of digital oral health care services there are several issues under licensure; the key issues are training, jurisdiction and scope of practice.
  - Training. The practitioner providing tele oral healthcare should be familiar with the technology (within a technology-evolving healthcare environment). For digital health to be effective, new technical skills, technologies and concepts may need to be learned by the oral health workforce [31]. This will require oral healthcare providers to engage in education and appropriate training. Dental education will need to prepare future oral healthcare providers to evaluate and interpret digital health systems. The oral health workforce must be exposed in a comprehensive manner to the foundational skills and knowledge needed to succeed as virtual health care practitioners [33]. Of course, as telehealth is a rapidly evolving field, courses should adapt to keep pace with changes in technology and best practices. Jurisdiction. Any health care activity has to comply with legal and ethical guidelines for the jurisdiction of practice. The use of Telehealth may cross jurisdictional borders (i.e., state, or international), which may create issues regarding licensure, for example.
    In some jurisdictions (e.g., Australia, Brazil, Europe), crossing state borders would not be an issue, and any licensed health practitioner could practice anywhere across the country. Still, health practitioners should always check with the regulatory authority for the profession in the jurisdiction where they intend to practice and in relation to any other requirements these bodies may have. As in other jurisdictions telehealth laws may be at the state, regional, or provincial level [29, 30].
  - Scope of practice. Advances in digital health create opportunities for some non-oral health professionals to act as primary intermediates between the main oral healthcare provider and the patient [34]. These providers may be very useful in tele oral healthcare.

2. Quality and standard of care. National Health Boards’ standards, codes, and guidelines regulate practitioners regarding quality of care when using telehealth to provide patient consultations/patient services, as they do when practitioners are delivering services face-to-face [7, 8, 35].
  However, although there are many instances where the use of telehealth is not different to face-to-face consultations and treatments, the use of digital health technology may introduce the possibility of some forms of negligence. It has been suggested that telehealth has the potential to increase the risk of misdiagnosis, bias and inequalities [36, 37]. For example, unless there are protocols for photographs, their quality may be affected and, consequently their interpretability and diagnosis [35]. Patients with limited or no digital literacy may face challenges in using telehealth successfully.
3. Patient - practitioner relationship. Health practitioners have traditionally focused on face-to-face interaction with patients. This relationship is based on mutual respect, trust and knowledge. Telehealth redefines this interaction because the communication is done through an electronic medium. Nonetheless, it is still covered by patient - practitioner privileges. In that sense e-communication (e.g., e-mail) can be perceived as being as private as face-to-face. However, the question arises whether e-communications can remain confidential or not (See point 6 and 8).
  This issue is also important because in some jurisdictions, malpractice applies only when a patient-practitioner relationship exists [30]. The interpretation of when such a relationship has been established with telehealth can vary on the nature of the healthcare services provided, or from one jurisdiction to another [30].
4. Consent. Obtaining informed consent is a prerequisite to providing any healthcare. Informed consent is defined as a person’s voluntary decision about health care that is made with knowledge and understanding of the benefits and risks involved [38].
  For informed consent to be valid, the patient must understand what they are consenting to [30]. This involves ensuring that information is provided to patients or clients in a way they understand, particularly, alternatives for consultation (e.g., face-to-face), fees, proposed treatment, sharing of information with others in the care team and whether there would be video or audio recordings of the consultation or not. Special considerations must be made when obtaining informed consent if the communication is not clear or fails during the consultation [39].
5. Authentication. Together with informed consent, a consultation also encompasses discussion of health professionals’ credentials and how they can be verified by patients (i.e., authentication) [18, 19, 30]. However, the process of authentication may be more difficult using telehealth, as there is no easy way to verify the credentials of who is providing advice [19, 30]. Patients should be made aware of this potential risk involved in teleconsultation.
6. Confidentiality and privacy. Confidentiality, and privacy are among the oldest and most important safeguards that the law offers to patients. They do not mean the same thing: Privacy (is the right of the patient), confidentiality (is the duty of the health provider). Privacy and data security have emerged as the most contentious issues in regard to use of digital health [3].
  A tele(oral)health consultation is clearly different to a face-to-face consultation regarding how data is handled. When parties are not in a common site for face-to-face consultation, there may be potential for breaches of privacy and confidentiality via data transmission [30]. Nevertheless, these responsibilities and duties do not change whether healthcare delivery is via telehealth or traditional face-to-face care [28].
7. Data security. From the legal perspective, the management (sanitisation, destruction, and disposal) of media on which digital health data is stored should be performed according to legislative obligations and sound technological practice. Secure storage remains the responsibility of the telehealth service provider [30, 40].
  As with privacy and confidentiality, accuracy, security, and integrity of data remain the responsibility of the telehealth service provider [28, 30, 40]. However, as mentioned, these changing professional roles may require additional training.
8. e-Referrals. Telehealth provides greater opportunity for multi-disciplinary care and enables certain monitoring tasks to be redirected to other health professionals, when appropriate. Oral health professionals, providing e-referrals, “the seamless exchange of significant patient information from one treating healthcare provider to another via a system of creating, storing and sharing referral reports” [41] should follow the necessary procedures [14]. Failure to refer a patient when required may constitute negligence. However, with digital health a referral could cross jurisdictions, which points to the need to review the standards of referrals [22]. Furthermore, even sending an e-mail about a patient to a colleague might be considered an e-referral [19].
9. Use of social media. Social media is clearly a topical part of our social fabric and also part of the digital revolution [1, 26, 31, 42]. There are many different types of social media, encompassing a wide range of websites and apps (e.g., Twitter, Facebook, LinkedIn, Instagram, Snap Chat, TikTok, etc.), which allow users to create and share content (i.e., short, or long written messages, photos, voice, and videos). Many platforms fit into more than one category, however, as the name implies, all are designed for social interactions [43].
  In addition to the various devices that can be used in telehealth, social media platforms are increasingly used for communication between oral healthcare providers and patients, follow-ups and advertising [31, 42, 44]. However, although they may have been described as offering “promising results” [37], they are not secure for clinical consultations or discussions, nor for file transfers. Images quality have been reported as inadequate with multiple sources of inaccuracy (e.g., reliability has not been fully established), which may end in underestimation of disease, for example [36, 37].
  Although social media, can be useful for organising follow ups, allowing communication by text messages, e-mails, or private messages, and can be used to disseminate general information, or for prevention and health education messaging, social media may not be adequate for any more personal, private information, which may need more protection [28, 45]. Thus, although there is no evidence that social media is heavily used as a platform for providing telehealth services, more work is required before it could be used for direct clinical consultations or discussions, or for transmission of patient files and health information [11, 12, 28, 31, 36, 44].
10. Financial and Remuneration issues. For telehealth to be effective, it must be integrated within the health services structure, with a dedicated annual budget, etc. Funding could be quickly implemented and then discontinued, if those services and programs are not “settled” into an organization and “embedded” in its normal activities (i.e., institutionalized). This is equally applicable across public healthcare and private practice.
  Regulations and guidelines are also important to organize the necessary financial resources for implementation and sustainability. Financing issues include not only reimbursement for the provision of health services, but also costs involved in the adoption of telehealth technologies. Introduction and maintenance of telehealth is associated with implementation and maintenance costs of technological infrastructure, as well as costs involved in learning technical aspects of using telehealth. Health practitioners without the finance or capabilities to provide telehealth may lag, creating additional access barriers to patients.
11. Adequacy of records. eHealth records, as with hard copy medical records, must provide the same comprehensive and accurate information. This ethical and legal responsibility does not change with the introduction of digital oral health. They are legal documents and have the same medico-legal risks relating to the management of hard copy dental records. As mentioned in Point 6, it is the healthcare provider’s responsibility to maintain the integrity, security, and confidentiality of eHealth records.
12. e-Prescriptions. E-Prescriptions must be handled very carefully [27]. Before proceeding to an e-prescription, during a teleconsultation, there must be confirmation of the diagnostics and procedures [27]. Additionally, any prescription must be done following guidelines for digital prescriptions, within the scope of practice and following strict individual evaluation (i.e., weight, age, etc.) [28].
13. Liability and malpractice. Most reviews warned about the potential for malpractice, negligence, and error during teleconsultation [30]. In fact, the conventional issues of malpractice and liability, are not different from face-to-face consultations. However, there is additional risk of liability and malpractice than for a face-to-face consultation [28, 30]. For example, it cyber liability may be an issue in case legal issues arise during data transmission [13, 28]. Golden and collaborators [30] suggest seeking advice from an experienced attorney.

## Discussion

In recent decades, and more so after the Covid-19 pandemic began, digital oral health has been increasingly used and telehealth services, technology and devices are fast developing. These tele oral healthcare services are not simply replacement for the existing modality of delivery (e.g., teleconsultation for patients/clients who cannot attend healthcare services), but a different way of using existing and new technology to provide oral healthcare, creating opportunities and features that were not available before [2]. Alongside, this growing use of tele (oral)health, there has been an expansion of guidelines, reviews, recommendations, reports, and research on ICT uses in almost all aspects of human life, including oral health.

Despite the positive impact of digital health on quality of care and its cost-effectiveness, several legal issues arising from these new practices have been identified [46–48]. Legal issues do not necessarily change whether healthcare delivery is via teledentistry or traditional face-to-face, the individual is still the patient, and with that come responsibilities and accountabilities. However, some technological issues may cause error, and although beyond the control of the practitioners, remain the responsibility of the practitioner [19, 26, 30]. Notwithstanding this, there is a general lack of discussion of legal issues around digital oral health.

Research in this field has mostly focused on the technical aspects of digital oral health, with legal, ethical and financial issues less covered. The current review indicated that the literature in this area has concentrated on familiar issue such consent, data privacy, licensing and jurisdiction, confidentiality, and patient-practitioner relationships. Results indicate that other legal issues (e.g., social media, authentication, e-referrals, e-prescription, remuneration and to a lesser extent, quality and continuity of care, and adequacy of records) are not receiving the same attention (See Table 3). Furthermore, in the present review, most of these issues were cited, but not described. Additionally, in some cases, they were only referred to in the context of specific dental disciplines (e.g., dental radiology, orthodontics) and not as broader issues across general dental practice. Thus, despite the good coverage shown in the present review, some issues (e.g., privacy, security, confidentiality, and patient-practitioner relationship) has not addressed adequately and may still need more scrutiny [3].

Sharing of pictures and images is not completely supported by accuracy (e.g., reliability has not been fully established) [25, 34]. Social media can be used as adjunct media, but may not replace clinical assessment or direct contact [36]. Additionally, for remote consultations using non-dental healthcare provider, there may be a need for more training programs in oral health issues. In addition, changes may be needed in the scope of practice of health professions to provide dental care beyond preventive treatments or using early detection assessments tools.

Conventional face-to-face consultation and treatment has well established legal procedures for patients to complain, and investigations to follow, but in regard to telehealth, the legal uses are not standardised or universal [7]. Postings on social media are legal documents [48, 49]. Around the world there are examples of inappropriate interactions that have cost those who posted. For example, recently two Melbourne dentists demanded the release of personal information from Google over a review and sued an anonymous patient for defamation [50]. This indicates that conventional mechanisms should and are still being used to complain.

Moreover, laws governing the disclosure of commercial agreements are not as clear as in other conventional media [26, 31, 51]. For example, social media does not have the same type of disclosure of any payment received for the promotion of products under the therapeutics goods advertisement code. Dental products advertised on Instagram were the subject of an investigation of the commercial manufacturer/distributor by the Australian Health Practitioner Regulation Agency [52, 53].

For digital health to be effective, new technical skills, technologies and concepts may need to be learned by the oral health workforce [31, 54, 55]. The future workforce should be able to evaluate and interpret the digital health technology that they will encounter in this environment. Thus, training in telehealth care should include more than just acquiring technological skills. Various barriers, both internal and external, may impact on the adoption and acceptance of this technology [56]. External barriers encompass tools, training, and support, while internal barriers involve attitudes, confidence, and beliefs in the need to incorporate technology [56]. In order to be effective, a telehealth curriculum should cover these barriers. Additionally, health professionals must be aware of the legal obligations, regulatory aspects, and funding considerations associated with telehealth [57]. Currently, there are reports that suggest that oral health practitioners are inadequately prepared for the implementation of teledentistry and face challenges in understanding privacy issues, obtaining informed consent, and ensuring record keeping, in digital health situations [11, 12, 25].

Tele(oral)health is quickly evolving and will continue to evolve. While technology can be implemented quickly, challenges are emerging in the development of best practices and norms for the use of these new and emerging digital technologies. Regulators need to keep pace with these changes. Most likely certain recommendations, guidelines, etc. may not be valid in the future due to changing and features of digital health platforms [28]. As with any technological advancements in health, new solutions will require regulations and a thorough evaluation of the intended use of the software and platforms involved. [28].

There is growing evidence that the therapeutic relationship between practitioner and patient when using telehealth can be as effective as in person care as well as evidence supporting patient satisfaction [58, 59]. However, by replacing face-to-face consultation, digital oral health disrupts some of the traditional norms which govern the health practitioner-patient relationship. For example, conveying oral health information in a clear way might be more difficult during telehealth consultations. Patients should be made aware of potential risks involved in teleconsultation. Confidentiality may also be threatened by e-communications, for example, the risk of computer hackers illegally accessing confidential information. An example of this would be the case of the hack of the largest health insurance provider in the world, Medibank [60]. In other scenarios an employee who has access to e-records may communicate information without a patient’s knowledge or consent, or an external technician may access patients’ information when conducting maintenance or repairs.

Thus, before implementing any digital health solutions, oral health practitioners need to be aware of the many legal challenges that the introduction of these technologies involves, be clear where the responsibility lies, and apply extreme caution in following national guidelines (e.g., General Dental Council [61]; Australian Health Practitioner Regulation Agency [8]). Oral health practitioners are recommended to familiarise themselves with the relevant guidelines, or even obtain legal advice, on the technological and ethical aspects of digital oral health [30] and remain aware of the legal requirements of the jurisdiction of practice. For example, at present, there are no well-defined regulations in place to address the legal and ethical issues that may arise due to the use of artificial intelligence and machine learning in health [62]. “Cloud” technology is another example impacted by lack of clear guidelines [63]. The enthusiasm to embrace the advantages that new technologies bring about, should not displace responsibilities for the ethical, social, and legal application of technology [64]. In this way, although legal issues have been discussed for years, they need to be revised and updated to address new challenges. This ought not be seen as a one-time event, but as an ongoing and iterative process. Continuous improvements to guidelines and laws will allow them to adapt and develop to keep pace with changes and challenges in technology and best practices in healthcare.

In the same way, past regulations need to be continually revisited, revised, and updated to ensure that the right to affordable access to digital technology is granted [13]. Laws and regulations are also meant to reduce inequalities in access to digital health, or the inability to use this technology (e.g., geographical, literacy, financial, connectivity, etc.) [4, 65], as well as to improve care to vulnerable populations [13]. The United Nations declared that human rights established within the physical world must also be established within the digital world, since Digital Rights are enablers for other rights [66].

Digital Health technology raises a range of legal questions and risks, many of which have been considered in the present review. Some of the limitations of this scoping review include the inherent lack of quantitative analyses of published studies and being prone to biases. This review could also have been more comprehensive by including countries legislation, however the purpose was not to reach exhaustion, but to reach saturation on the topic [3]. The range of legal issues presented in this review are intended as a starting point for further discussion of legal issues and responsibilities. In discussing them, we should not ignore the potential benefits of digital oral health. It is hoped that this review provides an appropriate and useful starting point for any oral healthcare practitioners as they embark on the digital health journey; as well as to other public health practitioners and managers, enhancing their practical understanding of the key legal, regulatory and financial aspects.

## Data Availability

All data generated or analysed during this study are included in this published article (Please see Tables 2 and 3).

## Abbreviations

ICT: Information and Communication Technology
EHR: e-Health Records
FDA: Federal Drug Administrating
LILACS: Literatura Latino-Americana y del Caribe en Ciencias de la Salud
